# Analysis of the influence of emotional factors on the efficacy and prognosis of endoscopic treatment of functional abdominal pain in children

**DOI:** 10.1097/MD.0000000000041741

**Published:** 2025-03-28

**Authors:** Lijun Cai, Wenying Yuan

**Affiliations:** a Department of Pediatric Gastroenterology, Maternal and Child Health Hospital of Hubei Province, Wuhan, Hubei Province, China; b Department of Pediatric, Maternal and Child Health Hospital of Hubei Province, Wuhan, Hubei Province, China.

**Keywords:** effect, emotional factors, endoscopic therapy, functional abdominal pain in children, prognosis, risk factor

## Abstract

This study aimed to explore the influence of emotional factors on the efficacy and prognosis of endoscopic treatment in children with functional abdominal pain (FAP), and to identify related risk factors. A total of 66 children with FAP treated with endoscopy from January 2018 to June 2024 were evaluated using the Child Depression Scale, Child Anxiety Scale, Quality of Life Scale, and visual analog scoring. Patients’ demographics, clinical symptoms, treatment methods, and outcomes were recorded. Symptom remission, recurrence rates, and quality of life changes were compared after 6 months. Pearson correlation and logistic regression analyses were conducted. High-anxiety (35 cases) and low-anxiety (31 cases) groups had mean pain scores of 4.85 ± 1.21 and 2.10 ± 0.85, respectively (*P* = .001). Recurrence rates were 34.3% and 9.7%, respectively (*P* = .012). Good-mood (20 cases) and bad-mood (46 cases) groups had quality of life scores of 85.50 ± 5.50 and 63.50 ± 7.00 (*P* < .05). High-depression (25 cases) and low-depression (41 cases) groups had mean pain scores of 5.10 ± 1.10 and 2.40 ± 0.75 (*P* < .05), with recurrence rates of 36.0% and 14.6%, respectively (*P* = .009). Anxiety and depression were positively correlated with pain scores (*r* = 0.60, *r* = 0.58, *P* < .05) and negatively correlated with quality of life (*r* = −0.56, *r* = −0.54, *P* < .05). Anxiety (OR = 3.20, *P* = .003) and depression (OR = 2.80, *P* = .007) were independent risk factors for recurrence. Emotional factors significantly affect the efficacy and prognosis of endoscopic treatment in children with FAP. Negative emotions increase recurrence risk and reduce treatment efficacy and quality of life. Psychological intervention should be considered to improve outcomes.

## 1. Introduction

Functional abdominal pain in children is one of the common diseases in pediatric clinic. According to statistics, about 20% to 40% of children in pediatric outpatient clinics will have recurrent abdominal pain symptoms, of which functional abdominal pain accounts for a considerable proportion.^[1]^ This disorder not only causes physical pain to children, but also has a negative impact on their daily life, learning and psychological development. Children are in a critical period of growth and development, and repeated abdominal pain can lead to problems such as poor concentration, decreased academic performance, and reduced social activity. From a clinical point of view, the diagnosis of functional abdominal pain in children usually requires the exclusion of organic disease. Although endoscopy and other means play an important role in the diagnosis process, physiological examination alone sometimes cannot fully explain the occurrence, development of diseases and the difference in treatment effect.^[2]^

Endoscopic therapy, as an important therapeutic method, has been gradually applied in the treatment of children with functional abdominal pain. It can directly observe the internal conditions of the gastrointestinal tract, and some potential lesions can be found and treated in time.^[3]^ However, in clinical practice, it has been found that despite the continuous technological progress of endoscopic therapy, the therapeutic effect varies greatly among different patients.^[4]^ Some patients have significantly relieved their symptoms after receiving endoscopic therapy, while others have poor results and even recurrent symptoms.^[5]^ This has led researchers to explore factors other than physiological changes. Among them, emotional factor as a potential influencing factor began to receive attention. A child’s emotional well-being is critical to their overall physical and mental health. Children face a variety of pressures and challenges as they grow up, such as learning pressure, family environment, social relationships, etc., which can affect their emotional state. Studies have shown that emotional problems such as anxiety and depression in childhood are not uncommon.^[6]^ Emotional problems such as anxiety and depression may affect the physiological functions of children through a variety of ways, including the neuroendocrine system and the immune system. Long-term anxiety may lead to disturbance of hormone levels in the body, which in turn affects gastrointestinal peristalsis and digestive function.^[7]^ In the context of functional abdominal pain in children, emotional factors may interact with gastrointestinal symptoms to aggravate the condition or affect the therapeutic effect.^[8]^ Pain perception is a complex physiological and psychological process. In children, emotional state has a significant impact on pain perception. Studies have found that children with anxiety and depression tend to be more sensitive to pain.^[9]^ This increased sensitivity to pain may be related to altered neural activity in areas of the brain associated with pain regulation. When children are in a state of anxiety or depression, the levels of neurotransmitters such as serotonin and dopamine in the brain will change, which may affect the transmission and perception of pain signals.^[10]^ In the case of functional abdominal pain in children, emotional factors may amplify the pain signal and make children feel more intense abdominal pain, thus affecting the effect of endoscopic therapy.

Children’s quality of life is an important indicator of their health status. There is a close correlation between emotional state and quality of life. Positive emotions help to improve children’s quality of life, while negative emotions can reduce it.^[11]^ For children with functional abdominal pain, bad mood will not only aggravate the symptoms of abdominal pain, but also affect their activities in daily life, social participation and mental health.^[12]^ Children who are in a bad mood may avoid physical activities and social gatherings because of abdominal pain, leading to a decline in their quality of life. The Child Depression Scale and the Child Anxiety Scale are commonly used tools to assess children’s emotional states. These scales have been studied and verified for a long time and have high reliability and effectiveness.^[13]^ In the study of functional abdominal pain in children, the use of these scales can accurately assess the level of depression and anxiety in children, providing a powerful tool for studying the relationship between emotional factors and diseases. Through the evaluation of a large number of children patients, the characteristics of functional abdominal pain in children under different emotional states and the differences in treatment effects can be better understood.^[14]^ Children’s quality of life scale is an important tool for evaluating children’s quality of life comprehensively. It covers many aspects of children’s life, including physical function, psychological state, social interaction, etc.^[15]^ When studying the influence of emotional factors on children’s functional abdominal pain, the children’s quality of life scale can help researchers observe the changes in children’s quality of life before and after treatment.^[16]^ By comparing the differences in children’s quality of life under different emotional states, the impact of emotional factors on children’s overall health status can be more deeply understood.^[17]^ Visual analog scoring is a simple and effective method for pain assessment. In children, this approach provides a visual indication of how much pain they feel.^[18]^ By asking children to mark their pain level on a straight line, pain assessment data can be quickly obtained.^[19]^ In the study of functional abdominal pain in children, visual analog scoring can be used to compare the severity of abdominal pain in children under different emotional states, providing a quantitative basis for the study of the relationship between emotional factors and pain.

In the past, there has been some exploration of the relationship between emotional factors and functional abdominal pain in children. Some studies have found that anxiety and depression are associated with the severity of symptoms of functional abdominal pain in children.^[20]^ However, most of these studies are based on small samples or single-center studies, and there is a lack of large-scale, multi-center research validation. Existing studies have not fully clarified the mechanism of how emotional factors specifically affect the effect and prognosis of children with functional abdominal pain endoscopic treatment. In terms of treatment, although some studies have proposed the importance of psychological intervention, further studies are needed on the specific methods, timing and combination with endoscopic therapy of psychological intervention. Therefore, this study aimed to explore the influence of emotional factors on the efficacy and prognosis of endoscopic treatment in children with functional abdominal pain (FAP), and to identify related risk factors.

## 2. Methods

### 2.1. Subjects

This study was approved by the Ethics Committee of Hubei Maternal and Child Health Hospital ([2024] IEC [038]). In our study, 66 children with functional abdominal pain who received endoscopic treatment in relevant medical institutions from January 2018 to June 2024 were selected as subjects. All patients or their guardians read the informed consent carefully and sign it. The hospital Ethics Committee approved the implementation of the study protocol.

#### 2.1.1. Inclusion criteria

The diagnostic criteria for functional abdominal pain in children were met, that is, abdominal pain caused by organic diseases was excluded after detailed history collection, physical examination, laboratory examination and necessary imaging examination. Age range is specified (6–14 years). Be able to cooperate with relevant scale evaluation and endoscopic treatment related operations.

#### 2.1.2. Exclusion criteria

Patients with organic diseases that may cause abdominal pain, such as gastrointestinal inflammation, ulcers, polyps, tumors, etc., have been confirmed by relevant examinations (gastroenteroscopy, abdominal ultrasound, CT, etc.). There are serious systemic diseases, such as heart, liver, kidney and other important organ failure, blood system diseases, etc., which may affect the safety and effectiveness of the study. Having a mental illness, such as schizophrenia or severe autism, can interfere with emotional assessment and the healing process. Recent (within 3 months) use of drugs that may affect gastrointestinal function or emotional state, such as certain antibiotics, antidepressants, anti-anxiety drugs, etc., unless discontinued long enough (as determined by the half-life and mechanism of action of the drug) to rule out the effects of the drug. Unable to complete the evaluation and treatment required for the study, such as intellectual retardation, severe behavioral problems.

#### 2.1.3. Treatment methods

All children who met the inclusion criteria received endoscopic treatment, and specific endoscopic treatment methods were selected according to the specific conditions of the patients and clinical routine operations, such as endoscopic observation of gastrointestinal mucosa, biopsy, polypectomy and other related operations. In the course of treatment, endoscopic procedures and asepsis principles are strictly followed to ensure the safety and effectiveness of treatment.

### 2.2. Evaluation index

The level of depression in children was assessed using the Child Depression Scale, which can effectively measure the severity of depressive symptoms in children. The children’s anxiety scale was used to evaluate the anxiety degree of children, and the behaviors and emotions related to children’s anxiety were reflected by each item. According to the evaluation results of the child depression Scale and the child anxiety Scale, the patients were divided into high anxiety group, low anxiety group, high depression group and low depression group.

The visual analogue Scale (VAS) was used to assess abdominal pain in children. Children were asked to mark their pain on a line ranging from 0 to 10, with 0 indicating no pain and 10 indicating the most intense pain.

Children’s quality of life was assessed by using the quality of life, which covers multiple dimensions such as physiology, psychology and society, and can fully reflect the overall state of children in daily life.

The patient’s symptom relief after treatment was recorded, including whether the abdominal pain was reduced and the frequency of attacks was reduced. The recurrence rate after treatment was measured, i.e. the proportion of patients with recurrent abdominal pain symptoms during the follow-up period.

Changes in quality of life before and after treatment were observed, and the effect of treatment on quality of life was assessed by comparing the differences in children’s quality of life scale scores.

### 2.3. Statistical methods

All statistical analyses were performed using SPSS 25.0 professional statistical software to check the completeness and accuracy of data. For continuous variables, such as age, visual analog score, total quality of life score, etc., the mean and standard deviation are described. For categorical variables, such as gender, anxiety and depression grouping, treatment effect, etc., the frequency and percentage were counted. Independent sample t test was used to compare the differences in visual analog score and total score of quality of life between high anxiety group and low anxiety group, high depression group and low depression group. Chi-square test was used to analyze the difference in recurrence rate between different anxiety and depression groups. Pearson correlation analysis was used to explore the correlation between anxiety level, depression level, visual analog pain score and total score of quality of life in children. Multivariate Logistic linear regression analysis was performed to determine whether anxiety level and depression level were independent risk factors for recurrence after endoscopic treatment of functional abdominal pain in children. In the regression analysis, other factors that might affect the results, such as age and gender, were also controlled. The significance level was set at *P* < .05 to determine whether the results were statistically significant.

## 3. Results

### 3.1. Comparison of visual analogue score and recurrence rate

As can be seen from Table 1, in terms of visual simulation score, the mean value of children in the high-anxiety group was significantly higher than that in the low-anxiety group. The *t* value obtained by independent sample *t* test was 7.987 and the *P* value was .000, indicating that children in the high-anxiety group felt more intense pain. In terms of recurrence rate, Chi-square test obtained an χ^2^ value of 6.304 and a *P* value of .012. The high anxiety group was also significantly higher than the low anxiety group, indicating that children with high anxiety were more likely to relapse after endoscopic treatment.

**Table 1:** Comparison of indicators between the high anxiety group and the low anxiety group

Group	N	Visual analog VAS score	*t*	*P* value	Recurrence rate	χ^2^	*P* value
High anxiety group	35	4.85 ± 1.21	7.987	<.001	34.3% (12/35)	6.304	.012
Low anxiety group	31	2.10 ± 0.85			9.7% (3/31)		

VAS = visual analogue scale.

### 3.2. The influence of emotional state on children’s quality of life

As can be seen from Table 2, the mean total score of quality of life of children in the good mood group was higher than that in the bad mood group. The *t* value obtained by independent sample *t* test was 17.258, and the *P* value was .000, which reflected that emotional state had a significant impact on children’s quality of life, and bad mood would lead to a decline in children’s quality of life.

**Table 2:** Comparison of the mean total score of quality of life between the good mood group and the bad mood group

Group	N	Quality of life score	*t*	*P* value
Good mood group	20	85.50 ± 5.50	17.258	<.001
Mood disturbance group	46	63.50 ± 7.00		

### 3.3. Comparison of various indexes between high depression group and low depression group

As can be seen from Table 3, the mean visual simulation score of children in the high depression group is higher than that in the low depression group. The *t* value obtained by independent sample *t* test is 9.765, and the *P* value is .000. Moreover, the recurrence rate obtained by Chi-square test is 6.832, and the *P* value is .009, which is also higher in the high depression group. This means that children with high levels of depression have more severe abdominal pain, as well as worse recurrence after endoscopic treatment.

**Table 3:** Comparison of various indicators between high depression group and low depression group

Group	N	Visual analog VAS score	*t*	*P* value	Recurrence rate	χ^2^	*P* value
High depression group	25	5.10 ± 1.10	9.765	<.001	36.0% (9/25)	6.832	.009
Low depression group	41	2.40 ± 0.75			14.6% (6/41)		

VAS = visual analogue scale.

### 3.4. Pearson correlation analysis

As can be seen from Table 4, Pearson correlation analysis shows that children’s anxiety level is positively correlated with visual analog pain score, that is, the higher the anxiety level, the higher the pain level. There was a negative correlation with the total score of quality of life, that is, the higher the anxiety level, the lower the quality of life. The level of depression in children was also positively correlated with the visual analogue score of pain and negatively correlated with the overall score of quality of life. Both *P* values were .000, indicating that both anxiety and depression were closely related to pain perception and quality of life in children.

**Table 4:** Correlation analysis of children’s anxiety, depression level and each index

Emotional factor	Correlation with visual analogue pain score (*R* value)	*P* value	Correlation with overall quality of life score (*R* value)	*P* value
Anxiety level	0.60	<.001	−0.56	<.001
Depression level	0.58	<.001	−0.54	<.001

### 3.5. Logistic multifactor linear regression analysis of independent risk factors

As can be seen from Table 5, after Logistic multivariate linear regression analysis, the OR values of anxiety level and depression level were 3.20% and 2.80, 95% confidence intervals were 1.50 to 6.80 and 1.30 to 6.00, and *P* values were .003 and .007, respectively. Wald values were 8.974 and 7.543, respectively, indicating that anxiety and depression levels are independent risk factors for recurrence after endoscopic treatment of functional abdominal pain in children, that is, rising anxiety and depression levels will increase the risk of recurrence after endoscopic treatment of functional abdominal pain in children.

**Table 5:** Logistic multivariate linear regression analysis results

Influencing factor	OR	95% CI	*P*	Wald
Anxiety level	3.20	1.50–6.80	.003	8.974
Depression level	2.80	1.30–6.00	.007	7.543

## 4. Discussion

The results of this study showed that the mean visual analog score of children in the high anxiety group was significantly higher than that in the low anxiety group (4.85 ± 1.21 vs 2.10 ± 0.85, *P* = .001), and the recurrence rate in the high anxiety group was also significantly higher than that in the low anxiety group (34.3% vs 9.7%, *P* = .012). This echoes the findings of some previous studies. Studies have found that anxiety can aggravate children’s perception of pain and negatively affect recovery after treatment.^[21]^ From the mechanism point of view, anxiety may affect the effect of endoscopic treatment of functional abdominal pain in children through a variety of ways. On the one hand, the dysfunction of autonomic nervous system in children under anxiety may lead to physiological dysfunction such as peristalsis and secretion of gastrointestinal tract.^[22]^ Anxiety, on the other hand, causes overactivation of pain-related neural circuits in the brain, making children more sensitive to abdominal pain. In this study, the level of anxiety in children was positively correlated with the visual analogue score of pain (*r* = 0.60, *P* < .05), further confirming the close relationship between anxiety and pain perception. Children with high depression had a higher mean visual analogue pain score than those with low depression (5.10 ± 1.10 vs 2.40 ± 0.75, *P *< .05), and had a higher recurrence rate (36.0% vs 14.6%, *P* = .009). This suggests that depression also has adverse effects on the efficacy and prognosis of endoscopic treatment for functional abdominal pain in children. The mechanism of depression affecting functional abdominal pain in children may involve neuroendocrine changes. When children are depressed, the levels of neurotransmitters such as serotonin and norepinephrine in their bodies change.^[23]^ These neurotransmitters play an important role in regulating gastrointestinal function and pain perception. In addition, depression may lead to changes in the way children perceive and cope with their illness, which can affect treatment effectiveness and prognosis. In this study, the level of depression in children was positively correlated with the visual analogue score of pain (*r* = 0.58, *P* < .05), and negatively correlated with the total score of quality of life (*r* = −0.54, *P *< .05), indicating that depression was closely related to pain and quality of life. The mean total score of quality of life in the good mood group (85.50 ± 5.50 points) was significantly higher than that in the bad mood group (63.50 ± 7.00 points, *P* < .05). This suggests that emotional factors have a crucial impact on children’s quality of life. Whether it is anxiety or depression, it may lead to the impairment of children’s functions in many areas such as physiology, psychology and society, thus reducing the quality of life. From the perspective of children’s daily activities, children with bad mood may reduce their participation in sports and social activities because of abdominal pain and negative emotions, which will affect their physical health and social development. In the family and school environment, emotional problems may lead to tension between children and their families, teachers, and classmates, further increasing their psychological burden. Logistic multivariate linear regression analysis showed that anxiety level (OR = 3.20, 95% CI: 1.50–6.80, *P* = .003) and depression level (OR = 2.80, 95% CI: 1.30–6.00, *P* = .007) was an independent risk factor for recurrence after endoscopic treatment of functional abdominal pain in children. This result clarified the importance of emotional factors in the prognosis of children with functional abdominal pain, and provided an important reference for clinical treatment. This means that in clinical practice, we cannot just focus on endoscopic therapy itself, but also need to evaluate and intervene in children’s emotional states. By reducing anxiety and depression levels in children, it is possible to reduce the recurrence rate of functional abdominal pain and improve treatment effectiveness.

Compared with previous studies, this study used a variety of assessment tools, including the child Depression Scale, the child Anxiety Scale, the child quality of life scale and visual analog scoring. The comprehensive application of these assessment tools enables us to more comprehensively and accurately evaluate the relationship between emotional factors and the outcome and prognosis of endoscopic treatment for functional abdominal pain in children. The study used only a single pain assessment tool, which may not fully reflect the overall condition of children.^[24]^ In comparison with previous studies, we found that this study was consistent with most research findings regarding the relationship between anxiety, depression and functional abdominal pain in children. Studies have also found that anxiety and depression can worsen the symptoms and prognosis of functional abdominal pain in children. However, there may be differences in some specific data and influence levels.^[25]^ This may be related to the selection of research objects, sample size, study area and other factors.

This study supplements and expands the previous research in the following aspects. First of all, we further verified the influence of emotional factors on the efficacy and prognosis of children with functional abdominal pain endoscopic treatment through a large sample size study, which increased the reliability of the study results. Secondly, we identified anxiety and depression as independent risk factors for recurrence after endoscopic treatment of functional abdominal pain in children, providing more specific intervention targets for clinical treatment. In addition, we also deeply explore the relationship between emotional factors and quality of life, providing new ideas for improving the overall health of children. Although this study adopted a more systematic study design, there are still some limitations. For example, this study is retrospective and there may be some recall bias. Although we collected as much detailed clinical data as possible during the study, we could not completely rule out the possibility of patients or family members having inaccurate recollection of symptoms. Our subjects were mainly from specific medical institutions, and there may be selection bias. These patients may differ in some way from patients in other regions or medical institutions, which may affect the extrapolation of the study results. In addition, there are some differences in the sample size of different groups in this study. Although corresponding treatment is carried out in the statistical analysis, it may still have a certain impact on the accuracy of the results.

In this study, we mainly focused on the influence of anxiety and depression on the efficacy and prognosis of endoscopic treatment for functional abdominal pain in children, but there may be other potential factors that were not included in the study. For example, family environment, social support and other factors may have an impact on children’s emotional state and disease prognosis.^[26]^ In addition, individual differences in children, such as personality traits and genetic factors, may also affect the study results to some extent. Based on the results of this study, clinicians should pay attention to the assessment of patients’ emotional state in addition to routine physical examination and diagnosis when they receive children with functional abdominal pain. Through the use of children’s depression scale, children’s anxiety scale and other tools, the emotional problems of patients were detected in time, and the emotional evaluation results were included in the formulation of treatment plans. Given that anxiety and depression levels are independent risk factors for recurrence after endoscopic treatment of functional abdominal pain in children, psychological intervention should be an important part of the comprehensive treatment of functional abdominal pain in children. The methods of psychological intervention can include cognitive behavior therapy, relaxation training, psychological support and so on. Studies have shown that cognitive behavioral therapy can effectively reduce the level of anxiety and depression in children and improve the symptoms of abdominal pain.^[27]^ The treatment of functional abdominal pain in children requires multidisciplinary collaboration. In addition to pediatricians and endoscopists, professionals such as psychologists and rehabilitation therapists should also be included. Multidisciplinary teams can work together to develop a personalized treatment plan that takes into account the child’s physiological, psychological and social factors to improve treatment outcomes. During the treatment process, the psychologist can be responsible for psychological intervention, and the rehabilitation therapist can guide the child to appropriate exercise and rehabilitation training. Improving the quality of life of children is one of the important goals in the treatment of children with functional abdominal pain. In addition to relieving the symptoms of abdominal pain, attention should also be paid to all aspects of the child’s daily life. For example, encourage children to actively participate in social activities, develop hobbies, and improve the family and school environment. These combined measures help alleviate children’s emotional problems and improve their quality of life.

Although this study has initially explored the mechanism by which anxiety and depression affect functional abdominal pain in children, there are still many unknown areas that need further research. How do emotional factors influence gastrointestinal function and pain perception through neuroimmune networks? Are there differences in the impact of different types of emotional problems (such as acute anxiety versus chronic anxiety) on functional abdominal pain in children? The research on these issues will help to deeply understand the relationship between emotion and disease, and provide more accurate theoretical basis for clinical treatment. In order to further verify the reliability of the results of this study, future prospective, randomized controlled studies are needed. By randomly assigning patients to different treatment groups (such as endoscopic therapy group and endoscopic therapy + psychological intervention group) and comparing the effects of different treatment methods, the role of psychological intervention in the treatment of functional abdominal pain in children can be more scientifically evaluated. At the same time, prospective studies can avoid problems such as recall bias in retrospective studies and improve the accuracy of research results. To make the findings more universally applicable, future studies need to expand the study to include children from different regions and socioeconomic backgrounds. At the same time, increasing the sample size can improve the statistical power of the study and more accurately detect the relationship between emotional factors and functional abdominal pain in children. In addition, special populations, such as children with other chronic diseases and children with a history of psychological trauma, can also be studied to explore the impact of these factors on children’s functional abdominal pain. In addition to anxiety and depression, there are many other factors that may interact with emotional factors to influence the treatment effectiveness and prognosis of functional abdominal pain in children. Family environment, social support, sleep quality, etc. Future research could explore the interaction between these factors and emotional factors and their combined effect on functional abdominal pain in children. Studies have found that the intensity of family environment is positively correlated with children’s anxiety level, and jointly affects children’s abdominal pain symptoms.^[28]^ Based on a deeper understanding of the mechanisms by which emotional factors affect functional abdominal pain in children, new treatments and interventions can be developed in the future. For neuroendocrine disorders caused by emotional factors, new drugs or biological therapies can be developed.^[29]^ At the same time, combined with modern technology, such as virtual reality technology, mobile health care, etc., can develop more innovative psychological intervention means. Virtual reality technology is used to simulate a relaxing environment to help children relieve anxiety and depression.^[30]^

Although this study provides valuable insights into the influence of emotional factors on the efficacy and prognosis of endoscopic treatment for FAP in children, several limitations should be acknowledged. First, the retrospective design may introduce recall bias, as the accuracy of symptom recall by patients or their guardians cannot be fully guaranteed, despite the collection of detailed clinical data. Second, as a single-center study, the generalizability of the findings may be limited, and future multi-center studies are needed to validate the conclusions. Additionally, imbalances in sample sizes across some groups may affect the robustness of the results. The study primarily focused on anxiety and depression, while other potential psychological factors (e.g., stress, trauma) were not explored, which may also influence treatment outcomes. The follow-up period was relatively short (6 months), limiting the assessment of the long-term impact of emotional factors on prognosis. Although variables such as age and gender were controlled, other confounding factors, such as family environment and socioeconomic status, were not fully accounted for. Furthermore, the study did not incorporate psychological interventions, and future research could explore the combined effects of endoscopic treatment and psychological interventions (e.g., cognitive-behavioral therapy). Finally, the reliance on self-reported scales may introduce reporting bias, and future studies could complement these with objective physiological measures. Despite these limitations, this study provides important references for the treatment and prognosis of FAP in children, and future research could further advance this field through improved study designs.

## 5. Conclusion

In our study, a variety of assessment tools and statistical analysis methods were applied to children with functional abdominal pain who received endoscopic treatment. It was found that the visual analog score and recurrence rate of the high anxiety and high depression groups were significantly different from those of the low anxiety and low depression groups. The level of anxiety and depression were positively correlated with the visual analog score of pain and negatively correlated with the total score of quality of life. Moreover, anxiety level and depression level are independent risk factors for recurrence after endoscopic treatment of functional abdominal pain in children, suggesting that emotional factors have a significant impact on the efficacy and prognosis of endoscopic treatment of functional abdominal pain in children. Clinical attention should be paid to emotional assessment and psychological intervention to provide new research ideas for endoscopic treatment of functional abdominal pain in children.

## Author contributions

**Conceptualization:** Lijun Cai, Wenying Yuan.

**Data curation:** Lijun Cai, Wenying Yuan.

**Formal analysis:** Lijun Cai, Wenying Yuan.

**Investigation:** Lijun Cai, Wenying Yuan.

**Methodology:** Lijun Cai, Wenying Yuan.

**Supervision:** Lijun Cai, Wenying Yuan.

**Writing – original draft:** Lijun Cai, Wenying Yuan.

**Writing – review & editing:** Lijun Cai, Wenying Yuan.
